# Hemodialysis pathway types influence wound healing complications and survival in end-stage renal disease patients in a retrospective cohort study

**DOI:** 10.1038/s41598-025-01602-1

**Published:** 2025-05-15

**Authors:** Jie Ding, Jun Li

**Affiliations:** Hemodialysis Ward, Wuhan Third Hospital, Tongren Hospital of Wuhan University, No. 216 Guanshan Avenue, Hongshan District, Wuhan, 430074 Hubei China

**Keywords:** Hemodialysis pathway, End-stage renal disease, Wound healing, Life cycle, Retrospective study, Nephrology, Kidney diseases

## Abstract

This study evaluates the impact of different hemodialysis access types—central venous catheter (CVC), arteriovenous graft (AVG), and autologous arteriovenous fistula (AVF)—on wound healing, complication rates, and long-term survival in patients with end-stage renal disease (ESRD). A retrospective analysis of 323 ESRD patients receiving hemodialysis over a ten-year period revealed significant differences in outcomes across the three groups. AVF patients experienced the shortest wound healing times and the highest dialysis efficacy, while the CVC group had the highest infection and reoperation rates. Although there were no significant differences in cardiac function or cause-specific mortality, AVF patients had the longest median survival time, followed by those in the CVC and AVG groups. These findings suggest that while AVF provides superior dialysis efficiency and survival outcomes with fewer complications, patient suitability and individual health conditions must be carefully considered when selecting the appropriate vascular access for hemodialysis.

## Introduction

Chronic kidney disease is acknowledged as a worldwide public health concern at this time^1^. Chronic kidney disease progresses to end-stage renal disease (ESRD) when functional nephron units gradually deteriorate, leading to a decline in renal function^2^. Worldwide, the number of ESRD patients is increasing by 7% annually^3^. According to the 2017 USRDS annual report, just in 2015, there were 500,000 individuals receiving maintenance hemodialysis and 124,111 new instances of ESRD^4^. Epidemiological surveys show that in 2012, among Chinese adults, the prevalence of chronic kidney disease was 10.8%, with the number of renal replacement therapy patients expanding at a yearly pace of 11%^5^. Currently, renal replacement therapy, primarily dialysis, is the most effective treatment for ESRD. Approximately 92% of patients undergoing dialysis receive hemodialysis, while around 8% undergo peritoneal dialysis. Thus, hemodialysis predominates as the renal replacement modality for ESRD patients. Globally, 1.5 million people receive hemodialysis treatment, with an annual mortality rate of from 10 to 25%^6^. In China, as of 2015, there were over 300,000 maintenance hemodialysis patients^7^. For ESRD hemodialysis patients, establishing and maintaining a well-functioning vascular access is crucial for ensuring smooth and adequate dialysis and is considered the lifeline of the patients^8^. However, current clinical use of vascular access still presents challenges, such as high mortality rates and high medical costs^9^. Clinically, hemodialysis uses three common types of vascular access: autogenous arteriovenous fistula (AVF), central venous catheter (CVC), and arteriovenous graft (AVG)^10^. Among these, AVF is the preferred dialysis method due to its adequate blood flow, fewer complications, and longer lifespan^2^. CVC can serve as an alternative for patients with poor vascular conditions or those who cannot tolerate AVF, particularly elderly patients with poor cardiac function or a short-expected survival period^11^. AVG is used for patients with poor vascular conditions, inadequate cardiac function, or those who cannot establish an AVF^12^. While there have been numerous clinical reports on the use of these three types of access in maintenance hemodialysis patients, studies on their impact on short-term wound healing, complication rates, and long-term survival in ESRD patients are limited. Thus, the purpose of this retrospective cohort research is to look at the effects of different hemodialysis access types (AVF, AVG, and CVC) on short-term wound healing, complication rates, and long-term survival in ESRD patients.

## Patients and methods

### Research flow chart

**Fig. 1:**
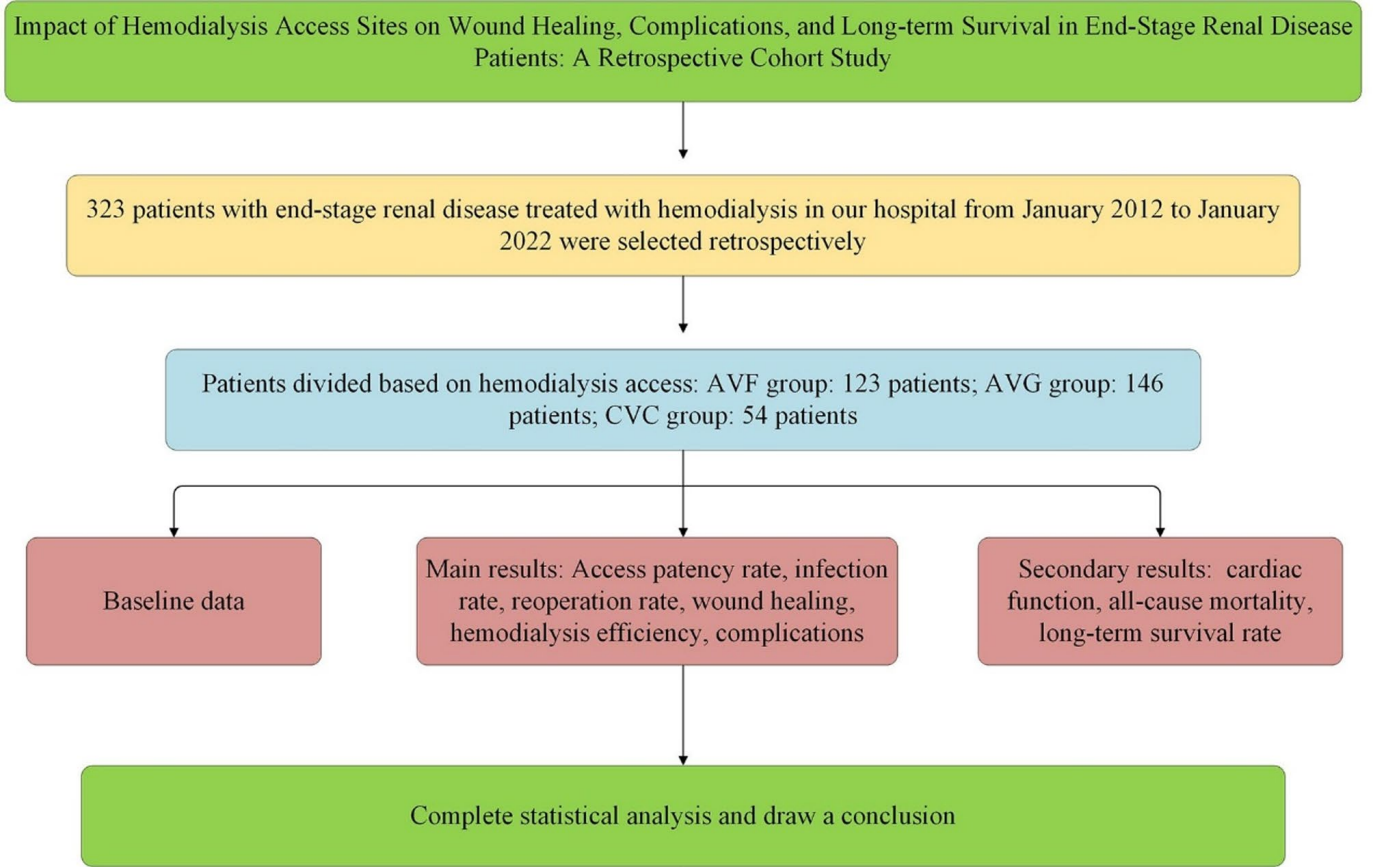
Research Flow Chart.

Figure 1 shows the flow chart of the research.

### Research objects

A retrospective selection was made of ESRD patients who underwent hemodialysis treatment from January 2012 to January 2022 in our hospital. A total of 323 cases were included in this research and categorized into three groups based on their hemodialysis access type: 123 individuals in the AVF group, 146 individuals in the AVG group, and 54 individuals in the CVC group. Patients with a history of chronic glomerulonephritis were included, and particular attention was given to those who had received immunosuppressive therapy. The duration and type of immunosuppressive agents used, as well as any associated complications, were documented.

### Inclusion/exclusion criteria

#### Selection criteria

1) Age: Patients aged 18 years or older.

2) ESRD, as all patients are on hemodialysis, which is an indication of ESRD.

3) Dialysis Duration: Patients with a history of ≥ 3 months of hemodialysis treatment.

4) Glomerular Filtration Rate: Patients with a glomerular filtration rate of ≤ 15 mL/min/1.73 m², consistent with the diagnosis of ESKD.

#### Exclusion criteria

1) Repeated registrants or serious data missing.

2) Patients with malignant tumors, acute and chronic inflammation, and significant problems with the heart, brain, lung, and other organs.

3) Patients with acute kidney injury undergoing temporary hemodialysis.

4) Patients who have not established long-term dialysis access or changed the type of long-term dialysis access due to various reasons (such as internal fistula occlusion).

### Ethical approval and method compliance

This study was approved by the Ethics Committee of the Wuhan Third Hospital. All methods were carried out in accordance with relevant guidelines and regulations, including the Declaration of Helsinki and institutional policies. Written informed consent was obtained from all participants prior to their inclusion in the study.

### Therapeutic methods

In the AVF group, an AVF was established using an end-to-side anastomosis of the cephalic vein and the radial artery. When under local anesthetic, a longitudinal or transverse incision was made 2–4 cm from the radial side of the patient’s wrist. The radial artery and cephalic vein were dissected and prepared, with ligation of the distal end. Next, an end-to-side anastomosis was carried out involving the cephalic vein and radial artery. This involved making a longitudinal incision of 0.6 to 0.8 cm in the radial artery, followed by continuous eversion and end-to-side suturing. Other venous branches near the anastomosis site were ligated to ensure proper venous return. Postoperatively, antibiotics were administered routinely to prevent infection.

In the AVG group, end-to-side anastomosis was performed with artificial polytetrafluoroethylene blood vessels. The forearm or upper arm brachial artery was selected as the artery end, and the median vein, noble vein, cephalic vein, and axillary vein were selected as the anastomotic blood vessels. After brachial plexus anesthesia, the veins and brachial arteries were separated. Artificial polytetrafluoroethylene vessels were implanted into the forearm volar side in a U-shaped or J-shaped configuration through a tunnel. The arteries and veins were then individually occluded or blocked. The vascular wall was longitudinally incised at a length of 0.4 to 0.6 cm at the fitting position, and the artificial vessels were continuously everted with the brachial arteries and veins. End-to-side sutures were then performed. Postoperatively, antibiotics were administered as routine practice.

In the CVC group, vascular access was established by puncturing either the femoral vein, subclavian vein, or internal jugular vein. Under local anesthesia, the modified Seldinger technique was employed. A needle from the disposable central venous catheter kit was used to puncture the vein. Once the needle entered the vessel, it was secured, and a guide wire was inserted to place the catheter. Subsequently, a single-needle, double-lumen venous catheter was inserted. This catheter was either temporary or tunneled, depending on the clinical indication, and the catheter was secured with three semicircular sutures. Finally, the puncture site was covered with a sterile dressing.

### Main results

(1) The dialysis patency rate (secondary patency rate, primary auxiliary patency rate and primary patency rate), wound healing time, infection rate and reoperation rate of the three groups were counted. We have now clarified the formulas for calculating the urea nitrogen reduction rate and clearance rate as part of the measurement of dialysis efficacy. The primary patency rate refers to the percentage of patients who have not undergone any corrective interventions (such as endovascular treatment or open surgery) within 12 months of follow-up and whose AVF remains patent. The primary assisted patency rate denotes the percentage of patients who did not experience fistula thrombosis or closure during the follow-up period, or whose fistula remained patent after interventions such as dilation or surgical correction of stenosis. The secondary patency rate indicates the percentage of cases where the fistula remained functional throughout the follow-up period or was successfully restored to patency after intervention, even if the catheter was temporarily removed. Wound healing was assessed based on the time required for the surgical incision site to heal completely, and the absence of infection or complications such as wound dehiscence. Wound healing time was recorded from the day of surgery to the complete closure of the incision, with follow-up evaluations at 1, 2, and 4 weeks post-operatively. Wound complications, including infection or delayed healing, were also monitored during these follow-up visits. The infection rate was defined as the percentage of patients who developed signs of infection at the surgical site during the follow-up period.

(2) Efficacy of vascular dialysis: Blood samples were collected from patients on dialysis one year after their treatment to measure parameters including urea nitrogen reduction rate, urea nitrogen clearance rate, and blood flow through vascular access channels. Urea nitrogen reduction rate = 100 × (1-C1/C₀), where C1 and C₀ represent urea nitrogen concentration before and after dialysis. Urea nitrogen clearance rate = In(R-0.008 t) + (4-3.5R) × UF/W, where UF is the quantity of ultrafiltration per dialysis, W is the patient’s body mass after dialysis, t is the time spent on each dialysis, and R is the ratio of urea nitrogen after dialysis to before dialysis. The blood flow through the vascular access was directly measured using the integrated flow sensor of the dialysis machine, which displays the effective blood flow rate in mL/min. This measurement method ensures a direct, reliable, and standardized determination of blood flow, thereby reducing potential variability associated with other measurement techniques (such as ultrasound).

(3) Complications such as thrombosis, internal fistula stenosis, infection, and ischemic syndrome were recorded postoperatively in both groups.

### Secondary result

(1) Cardiac function: The cardiac function of two groups was evaluated by color Doppler ultrasound before and 2 weeks after operation, including cardiac index, cardiac output and left ventricular ejection fraction.

(2) The number of all-cause deaths of patients were recorded in the two groups, including cardiovascular events, cerebrovascular events, infections, and other causes. All-cause death refers to fatalities resulting from any reason. Cardiovascular event death involves deaths where cardiovascular factors directly lead to conditions such as heart failure, myocardial infarction, arrhythmia, and sudden cardiac death. Cerebrovascular event death pertains to fatalities directly caused by cerebrovascular factors. Death due to infection indicates fatalities where infection is the primary cause of death.

(3) Statistics of three groups of patients with life cycle.

### Statistical analysis

Software called SPSS 21.0 was used to do statistical analysis. Measurement data were checked for homogeneity of variance and normal distribution before analysis to make sure they adhered to the normalcy or approach normality criteria. The findings are shown as mean plus standard deviation ($\overline{x}$± s). Repetitive measures analysis of variance was used to examine the repeated measures data. To evaluate group comparisons, the F-test was used. Using the χ² test, count data were evaluated and displayed as numbers and percentages (n [%]). Kaplan-Meier survival analysis was performed to produce survival curves, and the Log-rank test was used to assess group differences in survival rates. A P-value of less than 0.05 was found to be statistically significant.

## Results

### The clinical characteristics of patients

The age distribution of the patients in the AVF group was 65.45 ± 3.53 years, with ages ranging from 43 to 78 years. 49 females and 74 males were present. The average BMI was 22.18 ± 1.45 kg/m², with a range of 18.84 to 23.85 kg/m². There were 41 more instances, including 2 cases of polycystic kidney disease, 8 cases of hypertensive nephropathy, 37 cases of diabetic nephropathy, 35 cases of chronic glomerulonephritis, and other cases. Among these, patients with chronic glomerulonephritis had a history of immunosuppressive therapy, which was considered when analyzing their complications. Within the AVG group, which comprised 88 males and 58 females, with an average age of 65.58 ± 3.56 years, the ages ranged from 44 to 79 years. The BMI was 22.32 ± 1.67 kg/m² on average, with a range of 18.57 to 23.59 kg/m². The primary illnesses were 4 instances of polycystic kidney disease, 13 cases of hypertensive nephropathy, 41 cases of diabetic nephropathy, 45 cases of chronic glomerulonephritis, and 43 other cases. In this group, patients with chronic glomerulonephritis had been on immunosuppressive therapy for varying durations, and some developed related complications such as infections or graft dysfunction. The CVC group consisted of 20 females and 34 men, with an average age of 65.89 ± 3.58 years, with ages ranging from 43 to 77 years. The BMI was 22.75 ± 1.75 kg/m² on average, with a range of 18.15 to 23.72 kg/m². Twelve instances of chronic glomerulonephritis, fourteen cases of diabetic nephropathy, three cases of hypertensive nephropathy, one case of polycystic kidney disease, and twenty-four more cases were the primary illnesses. Among the CVC group, patients with chronic glomerulonephritis had a history of long-term immunosuppressive treatment and were monitored for complications such as bacterial infections or delayed graft function. The three patient groups’ combined general data lacked statistical significance.

### Comparison of dialysis patency rate, wound healing time, infection rate and reoperation rate

The comparison of primary patency rate, primary auxiliary patency rate, and secondary patency rate indicated that the AVF group was greater than the AVG group, which in turn was greater than the CVC group, with considerably differences (*P* < 0.001). The wound healing time was considerably shorter in the CVC group compared to the AVF group and AVG group (*P* < 0.001). The infection rate and reoperation rate were considerably higher in CVC group than in AVG group than in AVF group (*P* = 0.030). Table 1 displays all of the data findings.

**Table 1:** Comparison of dialysis patency rate, wound healing time, infection rate and reoperation rate among the three groups.

Group	*N*	Primary patency	Primary assistance is unobstructed	Secondary patency	Wound healing time(d)	Infection rate	Reoperation rate
AVF	123	96(78.05)	111(90.24)	118(95.93)	12.81 ± 2.33	6(4.88)	7(5.69)
AVG	146	78(53.42)	104(71.23)	104(71.23)	18.58 ± 3.18	18(12.33)	15(10.27)
CVC	54	25(46.30)	30(55.56)	29(53.70)	8.22 ± 0.94	13(24.07)	10(18.52)
*χ* ^*2*^ */F*		23.543	27.753	45.086	360.679	13.833	6.958
*P*		<0.001	<0.001	<0.001	<0.001	<0.001	0.030

Note: The numbers in parentheses represent percentages of the total cases for each category. χ² (Chi-square) test is used for categorical variables to compare frequencies between groups. F is the F-statistic from an ANOVA (Analysis of Variance) test, used to compare means for continuous variables between groups. AVF: Arteriovenous Fistula; AVG: Arteriovenous Graft; CVC: Central Venous Catheter. N: Number.

### Comparison of the efficacy of vascular dialysis

Based on the comparison of vascular dialysis efficacy, the AVF group demonstrated the highest urea nitrogen reduction rate, urea nitrogen clearance rate, and blood flow through vascular pathways when compared to the AVG and CVC groups. Specifically, the AVF group exhibited significantly higher values in these parameters, followed by the AVG group, and then the CVC group. Statistical analysis revealed significant differences in urea nitrogen reduction rate and urea nitrogen clearance rate (*P* < 0.001), and a marginally significant difference in vascular pathway blood flow (*P* = 0.015). Table 2 displays all of the data findings.

**Table 2:** Comparison of the efficacy of vascular dialysis in three groups of individuals [$\overline{x}$± s].

Group	*N*	Urea nitrogen decline rate(%)	Urea nitrogen clearance rate(%)	Vascular pathway blood flow(ml/min)
AVF	123	67.95 ± 3.95	1.69 ± 0.33	228.49 ± 24.95
AVG	146	66.95 ± 4.91^*^	1.59 ± 0.44^*^	222.91 ± 23.91^*^
CVC	54	63.91 ± 6.34^*#^	1.42 ± 0.22^*#^	217.75 ± 23.81^*#^
*F*		13.160	10.055	4.211
*P*		<0.001	<0.001	0.015

Note: Compared with AVF group, ^*^P < 0.05; Compared with AVG group, ^*#^*P* < 0.05. F is the F-statistic from an ANOVA (Analysis of Variance) test, used to compare means for continuous variables between groups. AVF: Arteriovenous Fistula; AVG: Arteriovenous Graft; CVC: Central Venous Catheter. N: Number.

### Comparison of the incidence of complications

The complication rate was highest in the CVC group, followed by the AVF group, and then the AVG group (*P* < 0.001). Table 3 displays all of the data findings.

**Table 3:** Comparison of incidence of complications involving the two groupings [n/%].

Group	*N*	Blood clots	Internal fistula stenosis	Infected	Ischemic syndrome	Total incidence rate
AVF	123	12(9.76)	5(4.07)	11(8.94)	6(4.88)	34(27.64)
AVG	146	10(6.85)	5(3.42)	4(2.74)	4(2.74)	23(15.75)
CVC	54	8(14.81)	2(3.70)	3(5.56)	3(5.56)	16(29.63)
*χ2*						7.225
*P*						<0.001

Note: The numbers in parentheses represent percentages of the total cases for each category. χ² (Chi-square) test is used for categorical variables to compare frequencies between groups. AVF: Arteriovenous Fistula; AVG: Arteriovenous Graft; CVC: Central Venous Catheter. N: Number.

### Comparison of cardiac function indexes

There weren’t any notable variations in cardiac index, cardiac output, and left ventricular ejection fraction among the three groups before and 2 weeks after the operation (*P* > 0.05). Table 4 displays every data result.

**Table 4:** Comparison of cardiac function indexes among three groups of individuals [$\overline{x}$± s].

Group	*N*	Left ventricular ejection fraction(%)		Cardiac output(L/min)		Cardiac index [L/(min·m^2^)]	
		Before operation	2 weeks after operation	Before operation	2 weeks after operation	Before operation	2 weeks after operation
AVF	123	50.81 ± 2.85	53.81 ± 2.45	4.71 ± 0.22	5.27 ± 0.32	3.19 ± 0.11	3.18 ± 0.66
AVG	146	50.59 ± 2.45^*^	54.11 ± 2.44^*^	4.74 ± 0.34^*^	5.20 ± 0.34^*^	3.15 ± 0.15^*^	3.21 ± 0.34^*^
CVC	54	50.82 ± 2.45^*#^	53.91 ± 2.55^*#^	4.78 ± 0.33^*#^	5.18 ± 0.34^*#^	3.17 ± 0.18^*#^	3.18 ± 0.32^*#^
*F*		0.292	0.510	1.068	2.029	2.650	0.152
*P*		0.753	0.748	0.349	0.133	0.074	0.859

Note: In contrast to AVF group, ^*^P > 0.05; In contrast to AVG group, ^*#^*P* > 0.05. F is the F-statistic from an ANOVA (Analysis of Variance) test, used to compare means for continuous variables between groups. AVF: Arteriovenous Fistula; AVG: Arteriovenous Graft; CVC: Central Venous Catheter. N: Number.

### Comparison of causes of death in ESRD

Based on the results in Table 5, there were no significant differences in the number of deaths caused by cardiovascular events, cerebrovascular events, infections, or other causes among the three groups (*P* > 0.05). However, there was a significant variation in the total number of deaths among the groups (*P* = 0.002), indicating a discernible difference in all-cause mortality. These findings are summarized in Table 5.

**Table 5:** Statistics on the causes of death of patients in two groups [n/%].

Group	*N*	Cardiovascular events	Cerebrovascular events	Infections	Other	Total
AVF	123	33(26.83)	27(21.95)	10(8.13)	48(39.02)	118(95.93)
AVG	146	38(26.03)	27(18.49)	8(5.48)	50(34.25)	123(84.25)
CVC	54	14(25.93)	10(18.52)	6(11.11)	18(33.33)	48(88.89)
*χ* ^*2*^		0.027	0.571	1.959	0.849	9.707
P		0.986	0.751	0.376	0.655	0.002

Note: The numbers in parentheses represent percentages of the total cases for each category. χ² (Chi-square) test is used for categorical variables to compare frequencies between groups. AVF: Arteriovenous Fistula; AVG: Arteriovenous Graft; CVC: Central Venous Catheter. N: Number. Total: total number of deaths.

### Comparison of life cycle of ESRD patients with different types of Hemodialysis pathway

**Fig. 2:**
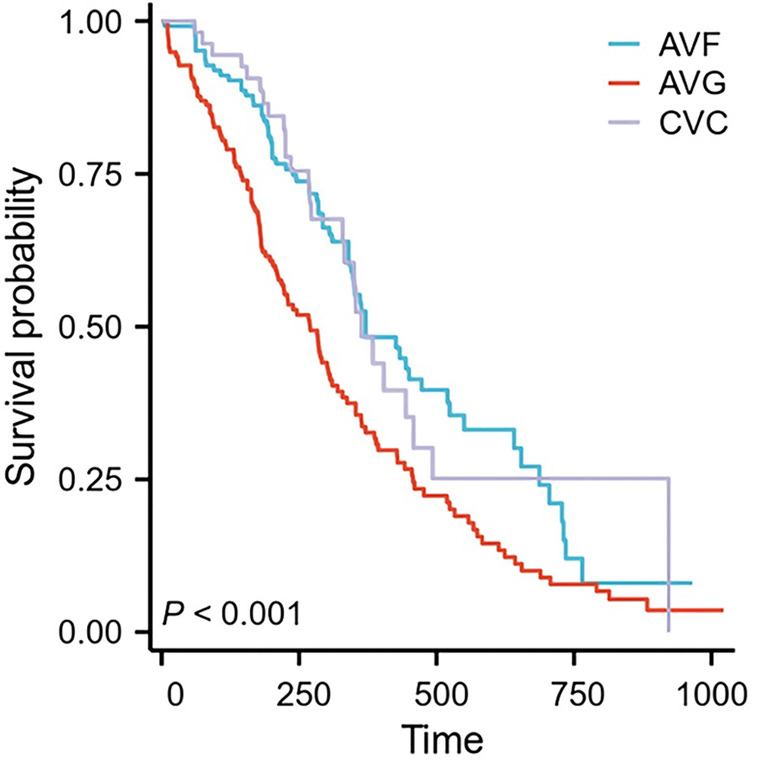
Comparison of life cycle of ESRD patients with different types of hemodialysis pathway.

The median survival time of the AVF group was 371 days [95% confidence interval (CI):348–524]. In addition, the median survival time of the AVG group was 352 days (95% CI:212–310). The median survival time of the CVC group was 363 days (95%CI:332–510, Log-rank = 16.052, *P* < 0.001, Fig. 2).

## Discussion

This study presents the results of a comparison of three commonly used vascular access types for hemodialysis in ESRD patients: AVF, AVG, and CVC. The key findings of this study show that AVF demonstrates superior dialysis efficacy, lower complication rates, and longer survival compared to AVG and CVC. Furthermore, while CVC was associated with the shortest wound healing time, it also had the highest infection and reoperation rates. These findings underscore the importance of selecting the most appropriate vascular access based on patient-specific conditions, taking into account not only dialysis efficacy but also the potential for complications and long-term survival. In particular, AVF showed superior dialysis efficacy, which can be attributed to its higher blood flow volumes and greater efficiency in urea nitrogen clearance. Our data reveal that, one year after treatment, the AVF group had markedly higher urea nitrogen reduction and clearance rates compared with both AVG and CVC (*P* < 0.001). To our knowledge, few, if any, previous studies have provided a direct comparison of these parameters among these vascular access types at the one-year mark. These novel findings underscore the superior toxin removal capability of AVF, which likely contributes to the improved survival outcomes observed in our study. The formula for calculating urea nitrogen reduction and clearance rates was included to further emphasize the effectiveness of AVF in removing toxins.

Maintenance hemodialysis is the primary treatment for individuals with ESRD, and vascular access is considered the “lifeline” for maintenance hemodialysis patients. Various vascular access types have been used in clinical practice, including AVF, AVG, and CVC, all of which differ in dialysis efficiency. This study shows that the patency rates, including primary patency rate, primary auxiliary patency rate, and secondary patency rate, were significantly better in the AVF group than in the AVG and CVC groups^13^. This supports the advantages of AVF in maintaining vascular access patency, which is likely due to its anatomical structure and compatibility with the vascular system. Although AVG and CVC have lower patency rates, they remain clinically significant^14^. We further clarified the differences between primary, secondary, and assisted patency, which are critical for understanding the long-term functionality of each access type. The CVC group exhibited the shortest wound healing time due to its superficial implantation, but it also had the highest infection and reoperation rates, highlighting the need for careful consideration when choosing vascular access. While AVF demonstrated superior dialysis efficacy, AVG and CVC can serve as necessary alternatives in certain clinical situations, such as in patients with poor vascular access or advanced comorbidities. AVF demonstrated superior dialysis efficacy and lower complication rates compared to AVG and CVC, making it the preferred option for long-term vascular access. However, the selection of vascular access should be individualized, based on a comprehensive evaluation of the patient’s condition. In patients with chronic glomerulonephritis or those receiving immunosuppressive therapy, the risk of infections and complications should be carefully considered, as these agents can increase the likelihood of catheter-related infections, particularly in the CVC group. This is not the first study documenting that the use of AVF is associated with longer survival. Previous studies, such as those by Ding et al.^15^, Li et al.^16^, also emphasize the superior survival outcomes associated with AVF. In patients with chronic glomerulonephritis or those who have received immunosuppressive therapy, careful attention should be given to the potential risk of infections and complications. Immunosuppressive agents may increase the risk of infection, particularly in the CVC group, which is more prone to catheter-related infections. As expected, differences in wound healing time were observed, with CVC requiring the shortest healing time due to its superficial implantation, while AVF required more extensive incisions, leading to longer healing times.

In ESRD patients, cardiovascular diseases account for over half of the deaths. In addition to the disease itself, hemodialysis places stress on the already compromised cardiovascular systems of maintenance hemodialysis patients^17^. The study revealed that the establishment of an AVF in maintenance hemodialysis patients led to a significant increase in left ventricular myocardial volume, which correlated with the blood flow rate^18^. The results indicated no significant differences in cardiac function parameters, such as cardiac index, cardiac output, and left ventricular ejection fraction, before and 2 weeks after the procedure. These findings suggest that vascular access (AVF, AVG, and CVC) did not have a significant short-term impact on cardiac function^19^. However, the study’s short observation period and limited sample size may have not captured the long-term effects of vascular access on cardiac function. Long-term hemodialysis patients are known to experience elevated cardiovascular morbidity and mortality, and the burden imposed by vascular access may exacerbate these outcomes.

In patients with chronic glomerulonephritis, especially those on immunosuppressive therapy, the cardiovascular risks may be even higher, given the combined effects of kidney disease, dialysis, and immunosuppressive agents. These patients may experience more pronounced cardiovascular complications, which could influence their long-term prognosis and survival. There weren’t any notable variations in the number of deaths attributable to cardiovascular events, cerebrovascular events, infection, or other causes among the three groups. However, when comparing the total number of deaths from all causes, a statistically significant difference was detected. Although there wasn’t a noticeable variation in mortality caused by cardiovascular, cerebrovascular and infection among the three groups, significant variations existed in their overall survival time. This suggests that the comprehensive effects of different vascular access types—especially in patients with underlying conditions like chronic glomerulonephritis and those on immunosuppressive therapy—could influence survival outcomes in ways that need to be further explored. The comprehensive effects of different treatments on patients, including the overall health status of patients, the effectiveness of treatment, the tolerance of side effects and so on. Therefore, in the selection of treatment, it is necessary to consider a variety of factors The comprehensive impact of different treatments on patients encompasses their overall health status, treatment efficacy, and tolerance of side effects. Therefore, treatment selection must consider multiple factors to enhance both the overall quality of life and life expectancy of patients. The median survival time was 371 days (95% CI: 348–524) in the AVF group, 352 days (95%CI: 212–310) in the AVG group, and 363 days (95%CI: 332–510) in the CVC group (Log-rank = 16.052, *P* < 0.001). The AVF group demonstrated a relatively longer median survival time compared to the AVG and CVC groups. This outcome likely reflects the stability and safety profiles of different vascular accesses over long-term use. Due to its unique anatomical structure and excellent vascular adaptability, the AVF group excels in maintaining vascular patency and enhancing dialysis efficiency. Consequently, patients in the AVF group can effectively eliminate metabolic wastes and toxins during long-term hemodialysis, thereby enjoying improved quality of life and living conditions. Conversely, the AVG group requires surgical implantation of artificial blood vessels, resulting in greater surgical trauma, longer postoperative recovery times, and a heightened risk of infection^20^. In the CVC group, direct insertion of the catheter into the body increases the risk of infection and catheter-related complications, thereby potentially impacting patients’ quality of life. Moreover, differences in the number of all-cause deaths underscore the importance of comprehensively considering patients’ overall health status, life expectancy, and the long-term safety of vascular access options^21^. For individuals with a long life expectancy and good physical condition, prioritizing AVF as the vascular access choice can lead to improved survival outcomes. In patients with chronic glomerulonephritis, the long-term effectiveness of AVF may be even more significant, considering the immunosuppressive therapy they often undergo. The need for careful monitoring of potential complications like infection and delayed healing is critical.

## Conclusion

In summary, AVF demonstrates superior dialysis efficacy, longer survival duration, and lower incidence of complications in hemodialysis. However, not all patients are suitable candidates for AVF treatment, especially those with poor vascular conditions, advanced age, or other serious comorbidities, for whom AVG or CVC may be more appropriate choices. Therefore, when selecting a vascular pathway, healthcare providers must thoroughly assess the patient’s overall condition and make decisions aligned with the patient’s preferences and expectations.

## Data Availability

The datasets used and/or analyzed during the current study are available from the corresponding author on reasonable request.
